# Case report: hikikomori syndrome in Italy and its link with autistic traits and internet gaming disorder

**DOI:** 10.3389/fpsyt.2024.1378572

**Published:** 2024-05-28

**Authors:** Barbara Carpita, Chiara Bonelli, Federico Giovannoni, Francesca Parri, Matteo Gambini, Benedetta Nardi, Giulia Amatori, Ivan Mirko Cremone, Stefano Pini, Liliana Dell’Osso

**Affiliations:** Department of Clinical and Experimental Medicine, Section of Psychiatry, University of Pisa, Pisa, Italy

**Keywords:** autistic spectrum disorder, internet gaming disorder, autistic traits, social withdrawal, hikikomori

## Abstract

During the last few decades, a growing field of literature is focusing on hikikomori, a phenomenon described as a form of pathological social withdrawal or social isolation that lasts for more than 6 months leading to significant functional impairment and/or distress. Despite initially considered a culture-bound syndrome, hikikomori syndrome later gained a wider recognition in different countries, ranging from an attempt to take refuge in an idealistic world, when society success’ standards are not reached, to a maladaptive coping strategy complicating several psychiatric illnesses such as anxiety disorders, major depression, internet addiction, internet gaming disorder (IGD) and autism spectrum disorder (ASD). In this framework, difficulties in social interaction, in problem solving strategies and socio-emotional reciprocity, may lead to social withdrawal and hikikomori-like behaviors. In this work, we described two cases of patients where the presence of underlying autism spectrum may have represented a sign of vulnerability towards the development of a possible full-blown case of hikikomori with IGD.

## 1 Introduction

*Hikikomori* is a Japanese term based on the combination of two words: *hiku* which means “to pull” and *komoru* which means “to withdraw” (1). This term became widely used in the second half of the 1990s (2, 3), after the publication of the book “Hikikomori – Adolescence Without End” (4), where it was used to describe “those who withdraw entirely from society and stay in their own homes for more than six months, with onset usually during the latter half of their twenties, and for whom other psychiatric disorders do not better explain the primary causes of this condition”. Initially, hikikomori was believed to be a phenomenon specific to Japanese society but, more recently, instances of this condition have been documented in several other nations, such as South Korea, France, Spain and the United States, and extensive coverage of it has appeared in worldwide media (5–13). The growing interest toward this condition also led to the proposal of two distinct categories of hikikomori: “*primary hikikomori*” where the social withdrawal is not caused by any comorbid psychiatric disorder, and “secondary hikikomori” where, on the contrary, social withdrawal can be attributed to a psychiatric disorder (14–16). A distinction was also highlighted between “hikikomori in the strict sense” (subjects who do not leave the house or leave it only for going to the convenience store, etc) and “quasi-hikikomori”, who would go out for pursuing their hobbies (17). In this context, the Italian version of the Hikikomori Questionnaire (HQ-25) (18) was recently developed and validated. The questionnaire features 25 items that investigate socialization, isolation and emotional support in suspected hikikomori subjects (19).

Despite the importance of this emerging condition, an agreement about the definition of hikikomori still has to be reached. It is commonly described as a pathological social withdrawal or social isolation that lasts for more than 6 months and causes significant functional impairment and/or distress (20, 21), not caused or influenced by an undergoing psychosis, and where some sort of social contact is not necessary but still allowed over the internet. Despite hikikomori was initially considered a culture-bound syndrome mainly relevant in Japan, it later gained a wider recognition as a syndrome that could be present also in different cultures in specific social conditions, characterized by the youths’ attempt to take refuge in an idealistic world when society success’ standards are not reached (1, 19). Recent literature further reconsidered hikikomori as a maladaptive coping strategy that could complicate several psychiatric illnesses and sometimes even be comparable to an alternative form of suicide attempt (1). In fact, hikikomori is now known to be comorbid with many psychiatric disorders including various anxiety disorders, major depression, personality disorders, internet addiction and internet gaming disorder (IGD) (10, 22–25). In particular, some authors highlighted how hikikomori and IGD may reinforce each other in a vicious circle, with hikikomori condition opening the way to the use of internet as a tool to communicate with the outside world and escaping towards videogames, while IGD would enhance the tendency towards social withdrawal for spending more time playing (26, 27). It has also been highlighted that hikikomori co-occurs in around one-third of those with autism spectrum disorder (ASD), which, in turn, has been associated with IGD (14, 25, 27–31).

Autism spectrum disorder (ASD) is a heterogeneous neurodevelopmental condition with varying degrees of symptom severity and with or without intellectual impairment. Its core symptoms are represented by pervasive impairment in reciprocal and social communication and interactions, restricted interests, repetitive, stereotyped behaviors, a lack of socio-emotional reciprocity, and impaired sensory integration processing (32). While ASD and hikikomori share some similar manifestations in the area of social skills, hikikomori is characterized in terms of social withdrawal, whereas ASD is defined in terms of social difficulties (33). In recent years studies examining how ASD presents in adults have progressively grown in number, with a particular focus on those mild manifestations without intellectual impairment that frequently remain under-diagnosed for many years (34, 35), coming to clinical attention only after the development of psychiatric comorbidities. Moreover, in the framework of a dimensional approach, sub-threshold autistic traits (AT) have been reported to be continuously distributed from the clinical to the general population is now generally acknowledged, probably sharing their genetic underpinnings with full-fledged clinical forms (36–39). It was also hypothesized that AT could be a vulnerability factor for different psychiatric conditions, also increasing the risk of suicidal ideation and behaviors (40–45). Noticeably, AT have been linked to a worse quality of life and a greater risk of suicidality, even at subthreshold levels (37, 45–48). Even though ASD and hikikomori may share some symptomatologic overlap such as deficits in social reciprocity and social withdrawal and, eventually, restricted and intensively pursued interests, to date the relationship between the two is still poorly investigated in the scientific literature (27). In this framework, it should be noted that a comorbidity with IGD has been reported for both conditions (27). Some studies highlighted that subject diagnosed with hikikomori reports higher AT, showing lower social skills and deficits in communication, imagination, attention span, multitask ability and adaptation to change compared to healthy controls (49), while ASD dysfunctional social interaction, causing deficiencies in introspectiveness and problem-solving strategies, may lead to a low self-esteem and social withdrawal (49, 50). In this work, we reported two cases of patients who manifested a severe social withdrawal comparable to western hikikomori-like syndrome, showing also significant autistic traits and IGD symptoms. Implications of the possible mediating role of AT in the development of hikikomori syndrome are discussed. Both subjects were given comprehensive information about the aim of this work and have been informed of the risks and benefits of allowing personal information to be used in this case report. Moreover, they were given the chance to ask questions prior to completing a written informed consent form.

## 2 Case presentation A

Mr. X.Y. is a 40-years-old, single South-American man who lives alone in Italy. He reported family history of psychiatric disorders (mother with major depression). Since childhood, he manifested panic-agoraphobic traits and social anxiety symptoms. Furthermore, he also showed relevant obsessive-compulsive traits with a tendency to order and precision, moral rigidity. Mr. X.Y. also displayed since childhood a behavioral pattern characterized by narrow and repetitive interests (a deep interest in manga and anime was maintained until present), strong adherence to routine, difficulties in socio-emotional reciprocity, empathy alterations, hyper-reactivity to sensory input, suggestive of an autism spectrum. Indeed, he described himself as an obstinate and stubborn child with few friends and who would have preferred study at home. He was not interested in other children’s games and had difficulties in establishing new friendships. He loved collecting objects and was extremely careful about his books and toys and did not like others to touch them. As a teenager, excelled in Maths and Physics, while showing poor performances in other fields. He spent the most of his spare time in solitary activities such as playing videogames. He was often teased by schoolmates or bullied during adolescence. Excelled in Maths and Physics, while showing poor performances in other fields.

The onset of clinical history was reported to be at the age of 15 when, subsequently to familiar economic troubles, with the need to move in another house, he developed swings in mood and energy and irritability, lack of motivation in studying, apathy, anhedonia and death thoughts. He did not seek medical help and maintained sufficient school and familiar functioning. At the age of 18, he moved to Italy and enrolled in bachelor program in Engineering. Soon after, he developed emotional lability, asthenia, anhedonia, lack of concentration, subsyndromal panic attacks with cardiorespiratory symptoms, anticipatory and performance anxiety, feelings of worthlessness and overwhelm, with inability to achieve regular academic standards and reduced interpersonal relationships. This clinical picture led to a progressive social withdrawal to the point of staying confined at home for about eight months without ever going out. He spent most his days studying in his bedroom, ordering food online with his only leisure activity being using internet especially for playing videogames. He interrupted contact with friends, contacted his family no more than once a week via phone and rarely maintained personal hygiene. He decided to return in South America, where, at the age of 20, he contacted a mental health expert and started a psychopharmacological therapy based on Sertraline up to 100 mg/day, with reported partial clinical benefit. Two years later, due to the presence of insomnia, Clonazepam was added to the therapy. Two years later, he moved again to Italy, but interrupted his studies and started his first job as seasonal employee and, for the persistence of the psychopathological symptoms described above, he seek again medical help, where he received the diagnosis of ‘Bipolar Disorder type II, depressive episode’ and was prescribed a psychopharmacological therapy based on Trazodone 15 mg/day, Fluvoxamine up to 200 mg/day (that was later replaced with Venlafaxine up to 150 mg/day) and Oxcarbamazepine up to 300 mg/day with partial clinical benefit. During the following years, the clinical picture improved, although he reported residual anxious and mood symptoms and a tendency towards social withdarawal. He went back to university and tried to engage in new social relations. In concomitance of the SARS-CoV-2 pandemic, Mr. X.Y. developed a dysphoric-irritable mood with emotional lability, decreased energy level and apathy, anhedonia, lack in concentration, intense rumination about its difficulties in maintaining social relations and suicidal thoughts, panic attacks, insomnia. Progressively he felt again pushed towards extreme social withdrawal, reducing contact with others to sporadic phone calls and going out when absolutely necessary (buy food, go to work in the months of employment) for about 1 year. Moreover, Mr. X.Y. increased progressively the time he spent on internet games. He also reported craving for high calorie food in the evening, with some binge eating episodes which led to an increasing of weight, reaching 121 Kg. After that, a therapy based on Melatonin 2 mg/die, Zopiclone 7.5 mg/day and Clomipramine up to 150 mg/day was recommended, determining a progressive improvement of affective symptoms and sleep, despite maintaining a tendency toward social isolation, going out from home only when necessary for work or for buying food, spending most of its spare time alone focusing on its specific interest about manga and anime, which he preferred not to share with other people.

One year later, the patient experienced again a decrease in mood, low energy levels, a worsening of social isolation and anxiety with hypersomnia and non-restorative sleep, avoidant and procrastinating behaviors, social withdrawal. Duloxetine up to 60 mg/day was introduced by the clinicians, together with perphenazine up to 2 mg/die, gradually reducing Clomipramine to 37.5 mg/day with reported partial improvement of well-being. During one of his most recent evaluations, Mr. X.Y. was assessed with the AdAS Spectrum questionnaire, in order to investigate underlying autistic traits, the Italian Version of the Hikikomori Questionnaire (HQ-25), and with the at assessment of Internet and Computer Game Addiction Scale (AICA CVS-S) for IGD. He reported a total score 89/160 at the AdAS Spectrum (35), over the validated threshold of 70 for possible clinically relevant ASD symptoms (35) (**Table 1**). On HQ-25, he reported a score of 84/100 (**Table 2**), above the threshold of 42/100 needed to underline an hikikomori condition (19), while at AICA CVS-S (51, 52) scale he reported a total score of 12.0, above the threshold of 7.0 for possible clinical symptoms of IGD (51) (**Table 3**). While the diagnosis of bipolar II disorder remained, it was updated underlining the presence of autistic traits and a tendency towards hikikomori-like social withdrawal and pathological gaming, now in remission.

**Table 1:** Case A AdAS score.

AdAS Domain	Score	N. items	Percentage
Childhood and adolescence	14	21	66,67%
Verbal Communication	11	20	55%
Non-Verbal Communication	12	28	42,86%
Empathy	6	12	50%
Inflexibility and adherence to routine	27	43	62,79%
Restricted interests and rumination	12	21	57,14%
Hyper-hypo reactivity to sensory imput	7	17	41,18%
Total	89	160	55,63%

**Table 2:** Case A, Case B HQ-25 score.

HQ-25 Domain	Score	N. items	Percentage
Socialization	36	100	36%
Isolation	24	100	24%
Emotional Support	23	100	23%
Total	84	100	84%
HQ-25 Domain	Score	N. items	Percentage
Socialization	29	100	29%
Isolation	24	100	24%
Emotional Support	11	100	11%
Total	64	100	64%

**Table 3:** Case A and B AICA CVS-S scores.

AICA CVS-S	Score	N. items
Case A	12.0	15
Case B	9.0	15

### 2.1 Case presentation B

Mr. W.Z. is a 20-year-old man who lives in a small town with his parents and sister. He reported no family history for psychiatric disorders. He described himself as shy and introverted, with social-phobic symptoms, including avoidance of public/school baths. He displayed narrow interests focused on computer and videogames, difficulties in interpersonal relationship, inflexibility, impaired social emotional reciprocity, hyper-reactivity to sensory inputs, ascribable to an autism spectrum.

During adolescence, at the age of 16, after a motorcycle accident, the patient began to experience swings in mood and energy, apathy and anhedonia, tendency to social withdrawal, panic attacks. The clinical picture worsened during the SARS-CoV-2 pandemic with a progressive avoidance of interpersonal relationships. He did not seek medical help and symptoms partially improved when returning to normal school life, although maintaining a pattern of difficulties in social relationships, with few and superficial friendships. During summer of 2022, in concomitance with his graduation exams, he experienced increased energy levels, irritability, decreased need for sleep. Few months later, following graduation and moving in another city for enrollment in a bachelor program in Informatics, he experienced difficulties in copying with the social and academic environment, with panic attacks and agoraphobic symptoms, ruminative thinking, mood decline, feelings of guilt, circadian rhythm dysregulation, insomnia. He progressively developed a complete social withdrawal with total interruption of relationships at the age of 20. While returning in his parents’ home during summer 2023, he spent most of his time closed in his room, going out only if pushed by his family for eating, avoiding even to use the bathroom, holding back urine or urinating in a bottle. The patient began to spend more and more hours on internet and online video games: he reported that the use of video games was his main distraction and link with other people. In august 2023, with the help of his family, he came to clinical observation. He reported a depressed mood with reduced energy and slight dysphoric notes, deep rumination on interpersonal experiences, feelings of self-worthlessness and guilt and anxiety elevation. He also reported pain and difficulties during urination, for which he was referred to an andrologist who made a diagnosis of prostatitis probably caused by excessive urine holding. He was treated with Valproic Acid 500 mg/die, Paroxetine 20 mg/die. The patient was also assessed with AdAS spectrum, HQ-25, AICA CVS-S questionnaires. He reported a total score of 96/160 on AdAS spectrum (35) (**Table 4**). On HQ-25 (19) he reported 64/100 (**Table 2**), while at AICA CVS-S (51) he reported a total score of 9.0 (**Table 3**). He received ae diagnosis of bipolar II disorder, panic disorder in a patient with autistic traits, and tendency towards hikikomori-like social withdrawal and IGD, now in remission. During the following months, W.Z. underwent a progressive improvement in symptoms, with a reduction in social withdrawal and resumption of university, while persisting the preference for solitary and household activities. He reported to not hold back urine anymore.

**Table 4:** Case B AdAS score.

AdAS Domain	Score	N. items	Percentage
Childhood and adolescence	15	21	71,43%
Verbal Communication	10	20	50%
Non-Verbal Communication	17	28	60,71%
Empathy	2	12	16,66%
Inflexibility and adherence to routine	24	43	55,81%
Restricted interests and rumination	15	21	71,42%
Hyper-hypo reactivity to sensory imput	9	17	52,94%
Total	92	160	57,5%

## 3 Discussion

### 3.1 Hikikomori syndrome and its link with ASD

We described two cases of a 40-year-old and of 20-year-old men with relevant autistic traits who, while displaying a number of autistic symptoms since early infancy, finally sought medical assistance after experiencing a severe hikikomori-like social withdrawal. As expected, given the comorbidity pattern of both autistic traits and hikikomori, the patients also manifested mood symptoms and traits of the IGD spectrum. During their infancy and later in life, Mr X.Y. and W.Z. manifested many autistic features that have represented the substance for the development of an affective disorder, a severe social withdrawal and a possible behavioral addiction.

During the clinical assessment, the AdAS Spectrum questionnaire was used, showing the presence of a high level of autistic traits, over the threshold for the presence of a full-blown ASD without intellectual impairment. In this framework, even though some studies highlighted a correlation between hikikomori and neurodevelopmental disorders (22, 25), the literature specifically focused on the relationship between hikikomori and ASD is still scant (49). A particularly consistent contribution to the field came from a recent study from Brosnan et al. (2023), carried on a sample of 646 subjects, that highlighted how autistic traits can strongly influence the risk of developing a form of hikikomori, also via mediating a poor psychological well-being (33). It also seems that poor social and problem-solving skills, typical of autistic subjects, can easily increase the risk of being involved in potentially traumatic events (53), such as bullying or peer rejection (49). This can lead to a reduced self-esteem and extra-punitive coping behaviors such as hikikomori-like social withdrawal (49). Furthermore, repetitive failures in the school and working environment, most easily found in autism spectrum subjects and lived as traumatic experiences, are strongly involved in the hikikomori condition (50). These evidences are all in line with Japanese studies that highlighted a link between hikikomori risk and higher levels of autistic features (29, 54).

Moreover, Mr. X.Y. and Mr. W.Z. both strongly manifested mood symptoms, associated with panic attacks and other anxious symptoms. This is in line with the evidence of a high rate of comorbidity between both ASD and hikikomori with many psychiatric disorders including anxiety and mood disorders (10, 22–26, 34, 42, 44, 55). In this context, low motivation, typical of depressive mental state, or the anxiety pattern in social situations typical of social phobia may favor the hikikomori state (56). Indeed, recent literature recognizes in what is defined as Modern Type Depression, some distinctives hikikomori traits, such as depressed mood, extrapunitive and strong avoidance tendencies, which may lead easily to social withdrawal (6, 57).

### 3.2 Hikikomori syndrome and its link with IGD

Another interesting aspect is represented by the fact that during their periods of social withdrawal, X.Y. and W.Z. reported a history of consistent internet gaming. During their clinical evaluation, they were assessed with the Italian version of AICA CVS-S scale reporting a total score higher than 7.0 points, which is clearly significative for the presence of IGD (57). Now added to the third section of the DSM-5 TR, IGD is described as a “persistent and recurrent use of the internet to engage in games leading to clinically significant impairment or distress and last up to 12 months” (32). For what concerns hikikomori state, the recent literature has introduced the Compensatory Internet Use (CIU) concept, defined as the refuge into the virtual world of Internet, which becomes a way to escape social deficits and a protective isolation against the outside world (26, 58, 59). Indeed, the use of internet games seems to be directly proportional to the prolongation of social withdrawal (60). In a similar way, the difficulties in establishing relationships and in social interaction, typical of the autistic dimension, could induce an increase in time spent on the internet through a pathologic social isolation (28, 30, 43). Moreover, ASD subjects are known to show marked reward dependency mechanisms and, therefore, are more easily exposed to behavioral addictions such as IGD (35, 36). On the other hand, the use of virtual communication can be seen as an attempt of adaptative behavior that can allow ASD subjects to present a completely different personality from themselves, appearing successful and socially acceptable (28, 61–63).

### 3.3 Hikikomori syndrome in Italy

Finally, it should be noticed that these are not the first cases of hikikomori-like social withdrawal in Italy (64). While hikikomori has been initially conceptualize as a culture-bond syndrome linked to the structure of Japanese society, it has been stressed how social changes in Western countries may open the way to the spreading of hikikomori outside Japan (65). In particular, difficulties in finding employment and marginalization due to economy stagnation led to an increase of the “Not in Employment Education or Training’ (NEET) phenomenon, especially in high-income countries, where there is a greater percentage of higher-educated people in spite of a decline of occupational possibilities. NEET and hikikomori share the tendency towards social isolation, unemployment, lack of sense of belonging. Interestingly, in both have been described a tendency to deviate from mainstream values and culturally common behaviors, which is also frequent among subjects in the autism spectrum (66). In this framework, NEET and hikikomori have been considered to be part of a same sectrum, with hikikomori at the extreme severe end (66).

In our case, in line with the literature available, the presence of an underlying autism spectrum may have represented a sign of vulnerability towards the development of a possible full-blown case of hikikomori with IGD tendency.

### 3.4 Treatment considerations

In both patients, the pharmacological treatment revealed its importance for the improvement of anxiety and mood symptoms, as well as for reducing social withdrawal and computer gaming time, leading to a global improvement of the well-being. In the first case, it required the inclusion of an antipsychotic drug. While patients did not follow a psychotherapy treatment, psycho-education about their conditions was provided during the clinical sessions with the psychiatrist, increasing the awareness about their symptoms and the compliance towards pharmacotherapy.

### 3.5 Limitations

This work should be considered in light of some limitations. First of all, the high number of questionnaire items. Despite HQ and AICA CVS-S are relatively short questionnaires, AdAS questionnaire requiries long compilation times increasing the risk of inaccurate and hasty answers. Conversely, large number of items allow us to explore a broad spectrum of symptoms. Moreover, all the questionnaires remain self-assessment that may lead patients to bias of symptoms’ overestimation/underestimation. Finally, the report of two patients limits general considerations about treatment indications. Moreover, patients were not assessed with quantitative psychometric instruments for anxiety and mood disorders, which were instead assessed towards a clinical evaluation according to DSM-5 criteria.

## Data availability statement

The original contributions presented in the study are included in the article/supplementary material. Further inquiries can be directed to the corresponding author.

## Ethics statement

Ethical review and approval was not required for the study of human participants in accordance with the local legislation and institutional requirements. The patients/participants provided their written informed consent to participate in this study. Written informed consent was obtained from the participant/patient(s) for the publication of this case report.

## Author contributions

BC: Conceptualization, Supervision, Writing – review & editing. CB: Writing – original draft. FG: Writing – original draft. FP: Writing – original draft. MG: Writing – original draft. BN: Supervision, Writing – original draft. GA: Writing – review & editing. IC: Writing – review & editing. SP: Conceptualization, Writing – review & editing. LD: Conceptualization, Supervision, Writing – review & editing.
